# Effect of a *Lactobacillus Salivarius* Probiotic on a Double-Species *Streptococcus Mutans* and *Candida Albicans* Caries Biofilm

**DOI:** 10.3390/nu9111242

**Published:** 2017-11-14

**Authors:** Wirginia Krzyściak, Dorota Kościelniak, Monika Papież, Palina Vyhouskaya, Katarzyna Zagórska-Świeży, Iwona Kołodziej, Beata Bystrowska, Anna Jurczak

**Affiliations:** 1 Department of Medical Diagnostics, Faculty of Pharmacy, Jagiellonian University Medical College, Medyczna 9, 30-688 Krakow, Poland; palina.vyhouskaya@mail.ru; 2 Department of Pediatric Dentistry, Institute of Dentistry, Jagiellonian University Medical College, Montelupich 4, 31-155 Krakow, Poland; dorota.koscielniak@uj.edu.pl (D.K.); iwona.kolodziej@uj.edu.pl (I.K.); anna.jurczak@uj.edu.pl (A.J.); 3 Department of Cytobiology, Faculty of Pharmacy, Jagiellonian University Medical College, Medyczna 9, 30-688 Krakow, Poland; monika.papiez@uj.edu.pl; 4 Department of Otorhinolaryngology, Jagiellonian University Medical College, Śniadeckich 2, 31-501 Krakow, Poland; katarzyna.zagorska-swiezy@uj.edu.pl; 5 Department of Toxicology, Faculty of Pharmacy, Jagiellonian University Medical College, Medyczna 9, 30-688 Krakow, Poland; beata.bystrowska@uj.edu.pl

**Keywords:** *Lactobacillus salivarius*, probiotic, *Candida albicans*, cariogenic biofilm, *Streptococcus mutans*

## Abstract

The aim of the study was to evaluate the anti-cariogenic effects of *Lactobacillus salivarius* by reducing pathogenic species and biofilm mass in a double-species biofilm model. Coexistence of *S. mutans* with *C. albicans* can cause dental caries progression or recurrence of the disease in the future. Fifty-nine children with diagnosed early childhood caries (ECC) were recruited onto the study. The condition of the children’s dentition was defined according to the World Health Organization guidelines. The participants were divided into children with initial enamel demineralization and children showing dentin damage. The study was performed on the *S. mutans* and *C. albicans* clinical strains, isolated from dental plaque of patients with ECC. The effect of a probiotic containing *Lactobacillus salivarius* on the ability of *S. mutans* and *C. albicans* to produce a double-species biofilm was investigated in an in vitro model. The biomass of the formed/non-degraded biofilm was analyzed on the basis of its crystal violet staining. The number of colonies of *S. mutans* and *C. albicans* (CFU/mL, colony forming units/mL) forming the biofilm was determined. Microorganism morphology in the biofilm was evaluated using a scanning electron microscope (SEM). In vitro analysis demonstrated that the presence of *S. mutans* increased the number of *C. albicans* colonies (CFU/mL); the double-species biofilm mass and hyphal forms produced in it by the yeast. *L. salivarius* inhibited the cariogenic biofilm formation of *C. albicans* and *S. mutans*. Under the influence of the probiotic; the biofilm mass and the number of *S. mutans*; *C. albicans* and *S. mutans* with *C. albicans* colonies in the biofilm was decreased. Moreover; it can be noted that after the addition of the probiotic; fungi did not form hyphae or germ tubes of pathogenic potential. These results suggest that *L. salivarius* can secrete intermediates capable of inhibiting the formation of cariogenic *S. mutans* and *C. albicans* biofilm; and may inhibit fungal morphological transformation and thereby reduce the pathogenicity of *C. albicans*; weakening its pathogenic potential. Further research is required to prove or disprove the long-term effects of the preparation and to achieve preventive methods.

## 1. Introduction

Early childhood caries (ECC) is one of the most widespread infectious diseases associated with biofilm formation in children worldwide—in Poland, it is prevalent in over 85% of pre-school children (<6 years) [1,2]. Untreated, ECC can lead to rapid tooth damage, causing pain and dangerous systemic infections [3]. Despite preventive measures and dental interventions, the first signs of ECC create a high risk of future recurrences [4], generating health and economic burdens among people at risk.

As proposed by the FAO/WHO (Food and Agriculture Organization of the United Nations, World Health Organization) definition, probiotics are live microorganisms, not causing any adverse effects on the organism and provide health benefits when administered in appropriate amounts [5].

Currently, interest in probiotic usage, in the case of caries prophylaxis, is growing, but their clinical efficacy in disease prevention appears limited and controversial [6,7]. Research on the use of probiotics in caries focuses primarily on their mechanism of growth inhibition and dental plaque reduction, created by pioneer *Streptococcus mutans* (*S. mutans*) strains, which are the main etiologic agent of this disease [8]. Even in clinical trials, endpoints usually constitute indirect measurements and are mainly associated with a decrease in *S. mutans* in the saliva [9,10,11] or a reduction of dental plaque acidity [12]. They do not affect the improvement of tooth enamel mineralization or the inhibition of plaque formation, which seems to be more adequate in the context of permanent (residential) colonization of *S. mutans* at the site of developing caries lesions (in plaque but not saliva). Bacteria, such as *S. mutans* as well as *Candida* genus fungi, present in the saliva (suspended as planktonic forms) are not direct etiologic factors of oral diseases [13]. Saliva is a material that only contains the transient presence of cariogenic bacteria or opportunistic fungi of the *Candida* genus and mediates the invasion of pathogenic bacteria in inflammatory foci [14]. 

In several clinical trials, the administration of *Lactobacillus* genus (*L. reuteri*, *L. salivarius*, *L. rhamnosus*) probiotics has demonstrated a decrease in the intensity of caries in children [10,15,16]. However, certain literature data show the reverse effects of the administration of *Lactobacillus*-containing probiotics (including *L. rhamnosus*, *L. reuteri*, and *L. paracasei*), due to the lack of *S. mutans* inhibition [17,18,19].

Probiotics contain microorganisms that do not show negative effects on the body; instead, they affect the human microflora through various mechanisms. They compete with other bacteria for nutrients and binding sites to the medium, inhibit their growth by producing bacteriocins, and further stimulate the immune response of the host. This occurs through the possible control of cytokine over-expression [20,21,22]. Nevertheless, the use of probiotics in immunocompetent hosts seems controversial, because of the reported cases of generalized infections from probiotic strains [20,23].

Indeed, *Candida albicans* (*C. albicans*) and *S. mutans* coexist for early childhood caries (ECC) [24,25]. On one hand, *Candida*-derived β-1,3-glucans affect the structure of the exopolysaccharide matrix (EPS), while mannan and β-glucan provide binding sites for glucosyltransferase B (GtfB). *Candida albicans* occurs in 96% of children with caries (age: 6–12 years), but only in 24% of children without this disease [26]. Currently, no in vivo studies have been conducted to demonstrate mutual interactions between *S. mutans* and *C. albicans* in models similar to actual oral cavity conditions in children with caries.

Oral streptococci produce proteins anchored in the cell wall to facilitate binding to *C. albicans* [27]. There is a specific hyperadditive effect of *S. oralis* and *C. albicans* during the fungi supported streptococcal biofilm production on mucous membranes [28]. Thus, not only mutants, but also species, such as *C. albicans*, can be decisive in determining the cariogenicity of the formed biofilms [29]. This was confirmed by recent studies, which demonstrated that increased *Candida* was associated with reduced diversity of salivary microbiota and displacement of the microbial aggregate toward streptococci [30].

Probiotics aggravate or delay the colonization of pathogenic bacteria during biofilm formation [31,32]. The mechanism of coaggregation of *S. mutans* with other bacteria has been intensively investigated, but there are few studies evaluating the effect of probiotics on this process. These focus mainly on the ability to inhibit mono-species biofilms or they evaluate the effects of probiotics only on salivary *S. mutants*, without evaluation of their relationships with an oral microbiome [33,34,35]. 

The limited number of studies on mutual interactions between clinical *S. mutans* and *C. albicans* strains in cariogenic biofilms and the effects of the probiotic *L. salivarius* on such interactions prompted us to address this problem.

The aim of the study was to evaluate the effect of a probiotic containing *Lactobacillus salivarius* on the mutual interactions of *S. mutans* and *C. albicans* as well as the ability to form a double-species biofilm, isolated from clinical strains, in an in vitro model. 

## 2. Materials and Methods 

### 2.1. Study Group

The examination was conducted according to the guidelines outlined in the Helsinki Declaration of 2008. The material was collected after the written consent of all participants (children and parents as their legal guardians). The Bioethics Committee of the Jagiellonian University in Krakow approved the study protocol (No. 122.6120.99.2016.).

The study was conducted from December 2016 to May 2017, involving a total of 59 pediatric subjects who had been screened/examined for study inclusion/exclusion by the University Dental Clinic, who were recruited by the Children’s Dentistry Laboratory of Dental Clinics, Jagiellonian University, Krakow. Bacterial strains were isolated from plaque samples derived from those participants (*n* = 59, mean age: 4.54 ± 0.79 years) who were diagnosed with early childhood caries (ECC) of the deciduous teeth. ECC was only diagnosed by a clinical examination.

The condition of the children’s dentition was determined in accordance with the guidelines of the World Health Organization for epidemiological studies on oral health, with the use of the International Caries Detection and Assessment System (ICDAS) classification. The study group was divided into two main groups: cavitated (where carious lesions were defined as cavity lesions in fissures and smooth surfaces with soft bottoms and walls) and non- cavitated (with white or brown enamel discolorations, but without enamel quantity damage, as well as undiluted enamel without cavities) [36,37]. 

The non-cavitated group (initial enamel demineralization, or white spots) corresponded to 1–2 in the ICDAS classification, whereas the cavitated group (dentine or cavitated damage) corresponded to 5–6 in the ICDAS classification [38]. Thirty participants qualified for the non-cavitated group, whereas 29 qualified for the group conventionally referred to as cavitated.

The exclusion criteria included: age below 2 years or over 6 years; inflammatory oral diseases, other oral diseases such as epithelial dysplasia, and periodontal pathology; and systemic illnesses, such as diabetes mellitus or hypertension. The use of antibiotics, anti-inflammatory drugs, or steroids, and a diet rich in supplements, such as vitamins or probiotics, in the past 5 months were also criteria for exclusion, along with partial or complete rejection of the dental examination by the child or their legal guardian. Plaque was evaluated, based on the simplified oral hygiene index (OHI-S index) [39].

### 2.2. Plaque Sampling Methods

Dental plaque was collected using dental probes after the patient’s qualification. Each patient was instructed about how to prepare for the test. Prior to the decision about giving consent for the study, details of the study and the scope of application were explained by the investigator. The study protocol was conducted with respect for the religious values of the study participants. The plaque samples were collected in the morning, between 8 a.m. and 9 a.m., under fasting conditions, and before the clinical examination and brushing. Prior to plaque collection, each patient rinsed their mouth with deionized water. Plaque samples were then placed in tubes containing 0.5 mL of phosphate buffered saline (PBS), maintained anaerobically. The samples were transported within 2 h at 4 °C to the laboratory. Plaque samples were broken up in an ultrasonic homogenizer (Hielscher UP50H) for 30 s at 25% amplitude and gently vortexed to give a homogeneous suspension. Cells were harvested in a logarithmic growth phase and washed 3 times with 40 mM potassium phosphate buffer (pH 7.0). Fifty μL of a homogenous microorganism suspension was used in the study for conventional culture methods, using a Sabouraud medium (Sabouraud Dextrose Agar, SDA) and a HLR-S (HL Ritz medium containing 40 g tryptic soy agar (TSA), 20% sucrose, 0.3 U/mL bacitracin, 1.75 μg/mL polymyxin B sulfate and 0.5 μg/mL crystal violet) selective medium, in three 10-fold dilutions, for each prepared sample. The material was incubated at 37 °C for 48 h under microaerophilic conditions (85% N_2_, 10% CO_2_, 5% O_2_). Positive samples were selected when colony numbers >10,000 cells/mL. The morphological characteristics of individual colonies cultivated on the medium with sheep blood were evaluated, as was the type of hemolysis caused by these colonies. In addition, dental plaque dilutions were performed, and these were inoculated on the Sabouraud medium and on the HLR-S medium. Once grown, the colonies were counted via determination of CFU/mL (colony forming units/mL). The number of microorganisms present in a particular test sample was determined using the formula:

CFU/mL = CFU × dilution factor × 1 aliquot

### 2.3. Characteristics of Isolated Species of Bacteria and Fungi

Pure *S. mutans* colonies were inoculated from the selective HLR-S to the Tryptic Soy Agar (TSA) with 5% sheep blood, as well as the Sabouraud medium, and incubated under the optimal conditions determined previously. The characteristic appearance of the colonies, including shape, form (single cells, pseudomycelium cells, or mycelium hyphae), hemolysis factor and other parameters that may create the phenotype were evaluated. Gram staining was performed every time as an element of a pre-differential diagnosis (gram-positive and gram-negative bacteria). 

### 2.4. Phenotyping

The species of isolated *S. mutans* were determined using a commercial STREPTOtest24 bioassay routine test (Erba Lachema, Brno, Czech Republic) and the API 20C AUX test (bioMérieux, Warsaw, Poland) for *C. albicans*.

### 2.5. Preparation of Microbial Suspensions

*S. mutans* and *C. albicans*, isolated from children with ECC, were used in the study. Single *S. mutans* and *C. albicans* colonies were cultured for 8 h at 37 °C in the presence of 5% CO_2_ in 4 mL of the Brain Heart Infusion medium (Merc, Darmstadt, Germany) and the Sabouraud liquid medium, with the addition of 5% sucrose, respectively. Bacteria and yeasts were harvested during the logarithmic growth phase, then washed twice with a 40 mM PBS (pH 7.0).

Microbial growth control was studied by flow cytometry (LSRII, BD Immunoassay Systems, San Jose, CA, USA). Cell conglomerates and doubles were discarded using a gated width-to-height spreading (FSC) and lateral scattering (SSC) strategy.

Bacterial and yeast suspensions were standardized to contain approximately 10^6^ CFU/mL. This was performed by dilution overnight of a bacterial/yeast culture in 5 mL of PBS. The inoculum density was measured using a MicroSpeak dual densitometer and confirmed by counting single colonies after 24 h growth under the same conditions as those described for *S. mutans* (Brain Heart Infusion Agar BHI agar) and *C. albicans* (Sabouraud dextrose agar).

### 2.6. Biofilm Generation

Biofilm generation was determined using a widely accepted, microtiter plate type, where the biofilm grew on the bottom and the walls of the wells, or on the disks located in the plate wells [40]. Clinical strains of *Streptococcus mutans*, selected as a pathogenic factor of caries and *Candida albicans*, as a potential ECC etiologic factor, were isolated from children with caries and used to form single or double-species biofilms, as described below. Biofilm formation occurred on polystyrene discs, placed vertically at the bottom of the 24-well microtiter plate wells, using sterile handles. Bacterial, fungal, and bacterial–fungal biofilm growth was investigated in the presence of a probiotic containing *L. salivarius* (HM6 Paradens). The crystal violet staining method was used to determine the biomass of the generated/degraded biofilm [41].

#### 2.6.1. Mono-Species Biofilm

**Figure 1:**
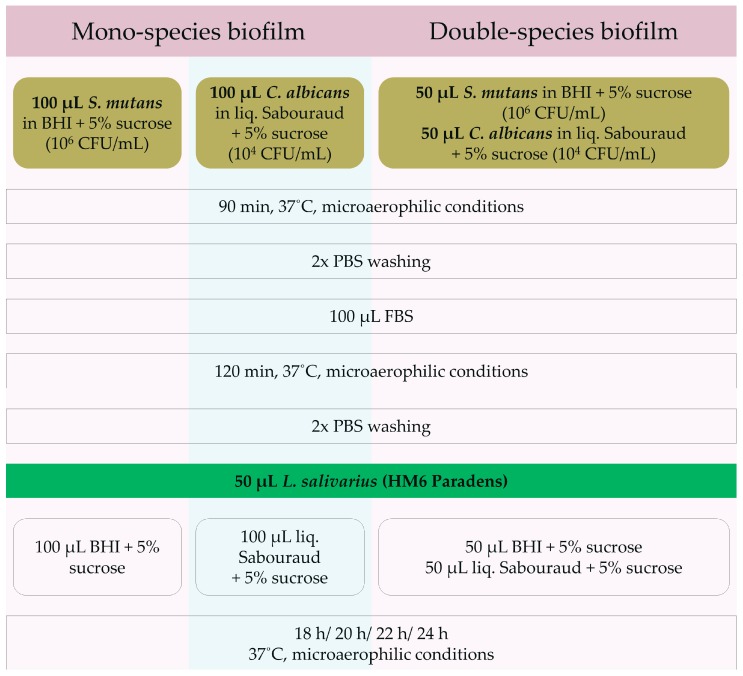
The protocol of biofilm generation and measurement. BHI: Brain Heart Infusion broth, CFU: colony forming units, liq: liquid, FBS: Fetal Bovine Serum, PBS: phosphate-buffered saline.

One hundred μL of a standardized bacterial suspension in a Brain Heart Infusion medium enriched with 5% sucrose of 1 × 10^6^ CFU *S. mutans*/mL density was added to the wells of the microtiter plate. One hundred μL of a standardized fungal suspension in a Sabouraud liquid medium with 5% sucrose of 1 × 10^4^ CFU *C. albicans*/mL density was added to the other wells. Plates were incubated for 90 min at 37 °C under microaerophilic conditions (85% N_2_, 10% CO_2_, 5% O_2_) to initiate the attachment of the microorganisms. Afterwards, the wells were washed twice with PBS solution. Next, 100 μL of FBS (Fetal Bovine Serum) was added to initiate biofilm formation, and the microplates were incubated for the next 2 h under microaerophilic conditions. Plates were then rinsed twice with PBS and 50 μL of a tested strain (HM6 Paradens) was added. Fifty μL of PBS (PBS Control), 50 μL of BHI + 5% sucrose (BHI Control), and 50 μL of Sabouraud + 5% sucrose (Sabouraud Control) were used as controls. For the biofilm growth and maintenance, 200 μL Sabouraud liquid medium with 5% sucrose or 200 μL of the BHI medium with 5% sucrose were added to *C. albicans* and *S. mutans* wells, respectively. Microplates were heated at 37 °C under microaerophilic conditions for 18, 20, 22, and 24 h (Figure 1).

#### 2.6.2. Double-Species Biofilm

In the case of double-species *S. mutans*/*C. albicans* biofilms, 50 μL of a standardized bacterial suspension in BHI medium enriched with 5% sucrose of 1 × 10^6^ CFU *S. mutans*/mL density and 50 μL of a standardized fungal suspension in a Sabouraud liquid medium with 5% sucrose of 1 × 10^4^ CFU *C. albicans*/mL density were added to the wells. Plates were incubated for 90 min at 37 °C under microaerophilic conditions (85% N_2_, 10% CO_2_, 5% O_2_) to initiate the attachment of the microorganisms. Afterwards, the wells were washed twice with PBS solution. Next, 100 μL FBS (Fetal Bovine Serum) was added to initiate biofilm formation, and the microplates were incubated for the next 2 h under microaerophilic conditions. Plates were then rinsed twice with PBS and 50 μL of a tested strain (HM6 Paradens) was added. Fifty μL of PBS (PBS Control), 50 μL of BHI + 5% sucrose (BHI Control), and 50 μL of Sabouraud + 5% sucrose (Sabouraud Control) were used as controls. For the *C. albicans*/*S. mutans* biofilm growth, 100 μL Sabouraud liquid medium with 5% sucrose and 100 μL of the BHI medium with 5% sucrose were added to the wells containing both *C. albicans* and *S. mutans*. Microplates were heated at 37 °C under microaerophilic conditions for 18, 20, 22, and 24 h (Figure 1).

Microorganism proportions in the wells were similar to those found in saliva samples in children with ECC. The organisms were grown without interruption, to allow generation and formation of biofilm, for 18, 20, 22 and 24 h, until the end of the experimental period.

### 2.7. Bacterial Enumeration (CFU/mL) in Biofilms 

The biofilms were washed thrice with phosphate-buffered saline at different time points (after 18, 20, 22, 24 h of incubation). The biofilm generated at the bottom of the well was removed by blending in an ultrasonic homogenizer (Hielscher UP50H, Teltow, Germany) for 20 s at 25% amplitude. Serial dilutions of the resultant solution were prepared and seeded in amounts of 100 μL on Sabouraud agar for *C. albicans* and mitis salivarius-bacitracin agar with sucrose MSBS (containing bacitracin and sucrose) for *S. mutans*, and then their growth was promoted for the next 48 h under microaerophilic conditions. The colony forming units (CFU/mL) were indicated. The protocol was performed in triplicate.

### 2.8. Biofilm Mass Determination

The mass of any formed biofilm was determined at different time points (after 18, 20, 22, 24 h of growth) via the crystal violet method. The formed biofilm was fixed in methanol (99+%, Sigma–Aldrich, Poznan, Poland) for 20 min. Then, the supernatants were discarded and the plates were air dried. Next, 125 μL of crystal violet (CV, 0.1%) solution was pipetted to microtiter plate wells. CV excess was removed by 3-fold PBS washing. The bounded stain was released with 200 μL of 95% ethanol (Sigma–Aldrich, Poznan, Poland). Subsequently, the contents of the wells were pipetted and 125 μL of the suspension was carried out to the new plate. The biomass of the generated biofilm was determined through its measured absorbance, using the standard curve at the maximum wavelength (λmax) = 540 nm. The protocol was performed at 20 to 25 °C twice at different times. The biofilm generation curve was plotted.

### 2.9. Scanning Electron Microscopic Analysis of Biofilm

Microbial biofilms were grown on 13 mm diameter round basic slides (Agar Scientific, Stansted, UK) in the wells of a 24-well plate, according to the protocol described above.

The slides were then stabilized in 1 mL of a 2.5% glutaraldehyde for 1 h and dehydrated in serial dilutions (50, 70, 80, 90, 95, 100% *v/v*) of ethanol for 20 min. Subsequently, the slides were immersed in 100% ethanol for 1 h. The slides were air-dried for one day and then transferred to copper disks and dusted with gold (160 s, 40 mA). The samples were analyzed using a scanning electron microscope (JEOL JSM-35CF, SEM, Jeol, Japan) at 20–25 kV in the Laboratory of the Otolaryngology Clinic, University Hospital, Krakow. The protocol was conducted at the above-mentioned time points in triplicate.

### 2.10. Statistical Methods

A statistical analysis was performed using R 3.2.3 (R Development Core Team, 2009). The ages of the children from the two subgroups were compared using Fisher’s exact test (because of the low expected numbers in the contingency table). The Shapiro–Wilk test was used to verify the normality of the data. Non-parametric analyses were conducted. Data are demonstrated as median and range. To compare CFU (log-transformed) and the optical density (OD) between *S. mutans*, *C. albicans*, and the co-culture, the Kruskal–Wallis test was performed. Dunn’s test (post-hoc test) was performed to determine which of the 3 groups actually differed.

The paired Wilcoxon’s test was used to compare CFU (log-transformed) and the optical density (OD), before and after *Lactobacillus salivarius* (HM6 Paradens) administration. Spearman’s coefficient of correlation was used to evaluate the relationship between colony-forming units (log-transformed) and the optical density (*p* < 0.05 indicated a statistically significant result).

## 3. Results

### 3.1. Study Design

During the study, bacterial and fungal strains were isolated from the plaque of children (*n* = 59, mean age: 4.54 ± 0.79 years) who were diagnosed with ECC of deciduous teeth.

Participants were assigned to two subgroups: children with early enamel deamination (white spots), defined as “non-cavitated” (*n* = 30; 1–2 in the ICDAS code); and children with dentin damage, assigned to the “cavitated” group (*n* = 29; 5–6 in the ICDAS code) [38]. Thirty participants, including 11 girls (4.91 ± 1.04 year/o) and 19 boys (4.47 ± 0.61 year/o), were allotted to the “non-cavitated” group, while 29 children to the “cavitated” one (14 girls aged 4.43 ± 0.94 years and 15 boys aged 4.47 ± 0.64 years). The form of caries did not depend on age, as shown in Table 1.

### 3.2. Morphological Characterization of Isolated Species of Bacteria and Fungi

**Figure 2:**
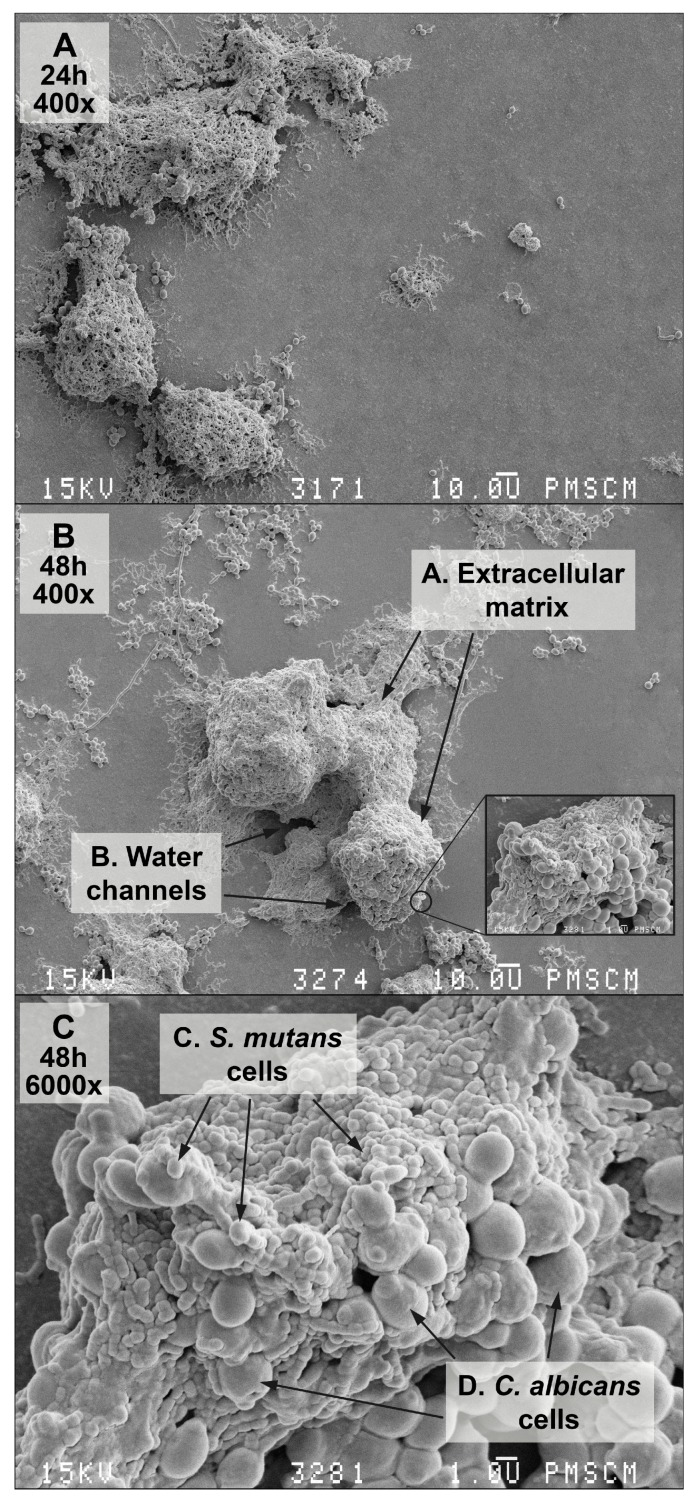
Scanning electron microscopy (SEM). (**A**–**C**) images of the double-species biofilm formed by *C. albicans* and *S. mutans*, after 24 (**A**) and 48 (**B**,**C**) h of biofilm formation. Culture was maintained at 37 °C, pH 7.0 and pCO_2_ 5%, in bovine serum as a medium additive promoting the growth of the culture in the presence of a sucrose substrate (5%). Original magnification: 400× and 6000×.

Interactions among *C. albicans* and *S. mutans* groups can create the biofilm demonstrated in Figure 2A–C. The isolated species of bacteria and fungi are listed in Table 2.

### 3.3. Analysis of Biofilm before and after Incubation with L. salivarius Probiotic

**Figure 3:**
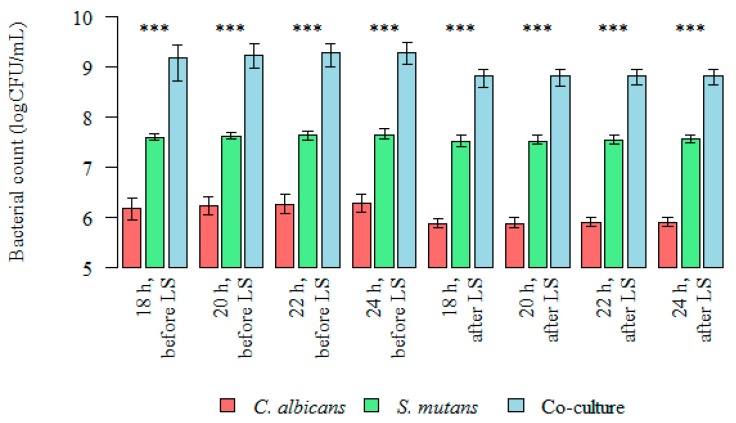
Intergroup differences between bacterial count (logCFU/mL), before and after *Lactobacillus salivarius* (HM6 Paradens, LS) administration. The Kruskal–Wallis test was used for dependent (repeated) measurements; *p* < 0.001; *** Dunn’s test (post-hoc test).

Differences in the colony forming units (CFU/mL) and the total biofilm mass of microorganisms (*C. albicans*, *S. mutans*, and the co-culture) at 18, 20, 22, and 24 h were statistically significant (Figure 3).

**Figure 4:**
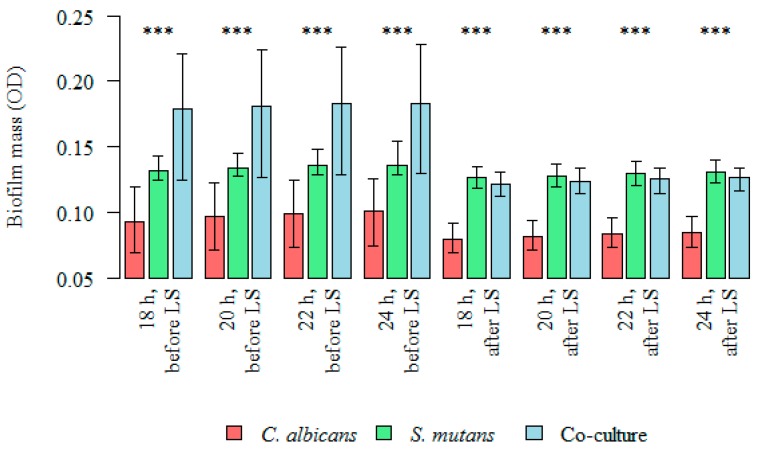
Intergroup differences between biofilm mass (OD), before and after *Lactobacillus salivarius* (HM6 Paradens, LS) administration. Kruskal–Wallis test was used for dependent (repeated) measurements; *p* < 0.05; *** Dunn’s test (post-hoc test).

**Figure 5:**
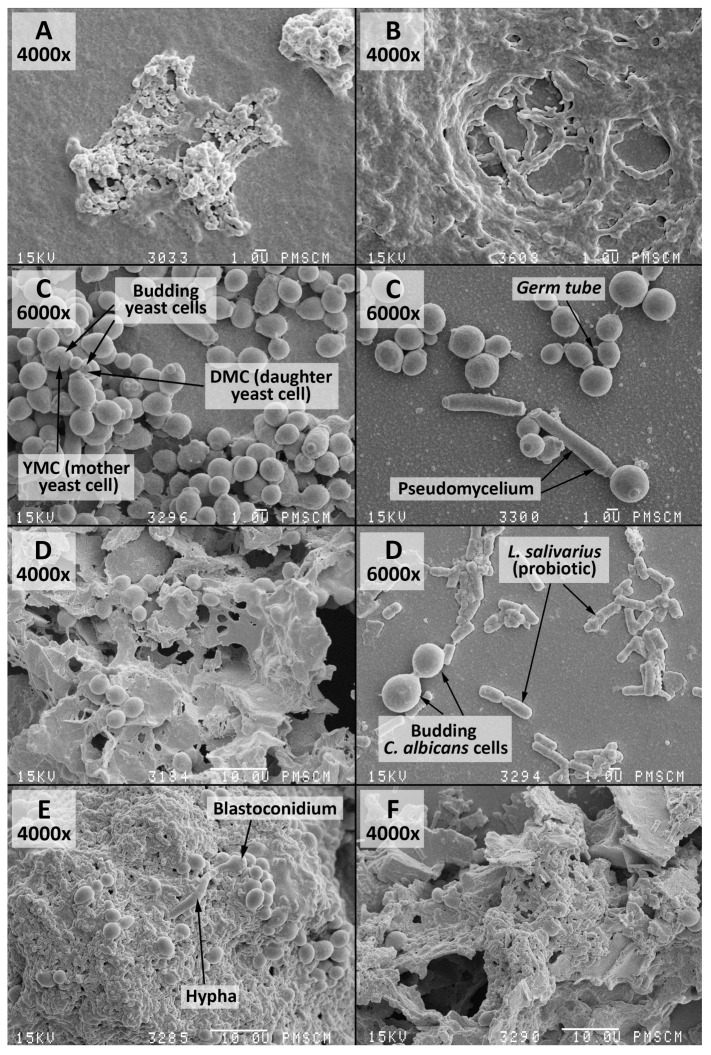
SEM images of the mono-species biofilm, generated by *S. mutans*, *C. albicans* and the double-species oral streptococci/yeasts biofilm—untreated and treated with the *Lactobacillus salivarius* (HM6 Paradens)—after 24 h of biofilm formation. (**A**) The 14 h *S. mutans* biofilm formed on a flat agar surface (Agar Scientific, Stansted, UK). *S. mutans* adheres to the polystyrene surface, mainly using a sucrose-dependent mechanism; (**B**) The 24 h *S. mutans* biofilm formed on a flat agar surface; visible polymeric Extracellular Matrix (ECM), which has an open architecture with nutrient channels, and other properties; (**C**) The 24 h *C. albicans* biofilm formed on a flat agar surface (Agar Scientific, Stansted, UK). *C. albicans* adheres to the polystyrene surface, mainly using mycelial forms, visible pseudohyphae, budding yeast, and the so-called germ tube, considered to be the key features of the pathogenicity of the fungus. The culture was maintained at 36 °C, pH 7.0 and pCO_2_ 5%, in bovine serum as a medium additive promoting growth of the culture in the presence of a sucrose substrate (5%); (**D**) The 24 h *C. albicans* biofilm formed on a flat agar surface (Agar Scientific, Stansted, UK) under the influence of *Lactobacillus salivarius* (HM6 Paradens). There was no clear, compact structure for the *C. albicans* biofilm and single loosely located budding cells. Other morphological forms of yeasts were invisible; (**E**) The 24 h double-species oral streptococci/yeasts biofilm formed on a flat agar surface (Agar Scientific, Stansted, UK). There was an apparent change in the *C. albicans* morphotype in the *S. mutans* common culture and visible pleomorphic forms were true hyphae and, blastoconidia, which in the mixed culture also produce mycelial forms, whose role is related to damage to immune cells (macrophages), leading to microorganism invasion. There was abundant extracellular matrix between cells and covering bacterial and yeast cells. Bacterial cells were visible in chains adhering to yeast cells and wrapped around them; (**F**) The 24 h double-species *S. mutans*/*C. albicans* biofilm formed on a flat agar surface (Agar Scientific, Stansted, UK) under the influence of *Lactobacillus salivarius* (HM6 Paradens). There was no clear, compact *S. mutans*/*C. albicans* biofilm structure or single loosely located budding cells. Other morphological forms of *C. albicans* were invisible. There was no visible extracellular matrix. Original magnification: 4000× and 6000×.

**Figure 6:**
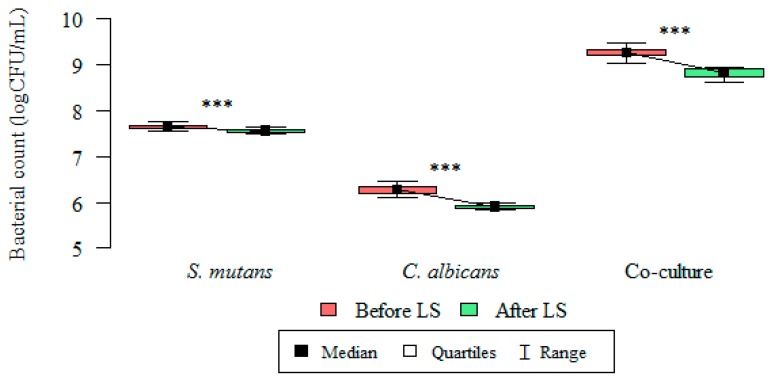
*S. mutans*, *C. albicans*, and oral streptococci/yeast biofilm formation: changes in microorganism count (logCFU/mL), before and after administration of *Lactobacillus salivarius* (HM6 Paradens) after 24 h. Wilcoxon’s test was used for dependent (repeated) measurements (paired Wilcoxon’s test; *p* < 0.05). Data are represented as median ± 1–3 quartiles for the three experiments. *** Indicates statistically significant difference compared to untreated biofilms (*p* < 0.001).

**Figure 7:**
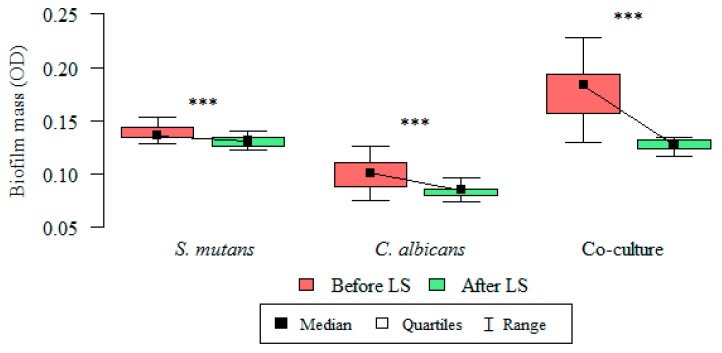
*S. mutans*, *C. albicans*, and oral streptococci/yeast biofilm formation: changes in optical density (OD) of biofilm mass, before and after administration of *Lactobacillus salivarius* (HM6 Paradens) after 24 h. Wilcoxon’s test was used for dependent (repeated) measurements (paired Wilcoxon’s test; *p* < 0.05). Data are represented as median ± 1–3 quartiles for three experiments. *** Indicates statistically significant difference compared to untreated biofilms (*p* < 0.001).

**Figure 8:**
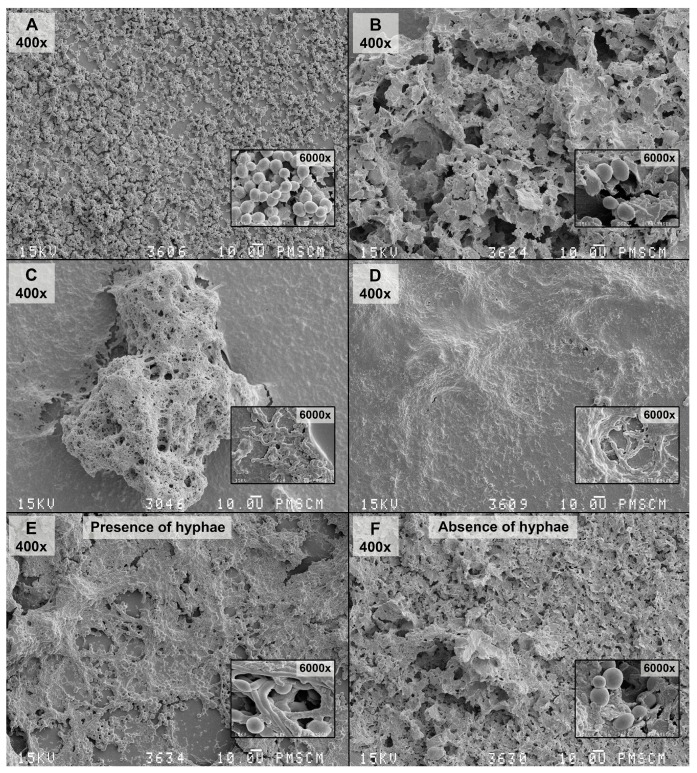
SEM images of the mono-species biofilm generated by *C. albicans*, *S. mutans* and the double-species oral streptococci/yeasts biofilm—untreated and treated with the *Lactobacillus salivarius* (HM6 Paradens)—after 24 h of biofilm formation. (**A**) The 24 h *C. albicans* biofilm formed on a flat agar surface (Agar Scientific, Stansted, UK); (**B**) The 24 h *C. albicans* biofilm formed on a flat agar surface treated with the *Lactobacillus salivarius* (HM6 Paradens); (**C**) The 24 h *S. mutans* biofilm formed on a flat agar surface (Agar Scientific, UK); (**D**) The 24 h *S. mutans* biofilm formed on a flat agar surface treated with the *Lactobacillus salivarius* (HM6 Paradens); (**E**) Co-culture oral streptococci/yeasts biofilm—untreated with the *Lactobacillus salivarius* (HM6 Paradens)—after 24 h of biofilm formation; (**F**) Co-culture oral streptococci/yeasts biofilm—treated with the *Lactobacillus salivarius* (HM6 Paradens)—after 24 h of biofilm formation. The Culture was maintained at 36 °C, pH 7.0 and pCO_2_ 5%, in bovine serum as a medium additive promoting growth of the culture in the presence of a sucrose substrate (5%). The occurrence of a co-culture biofilm at this stage may depend on the *C. albicans* morphotypes showing a twofold nature: buds and *C. albicans* hyphae may colonize mucous membranes and constitute physiological microflora (commensal) or may lead to infection under favorable conditions (opportunistic pathogens). There was no clear, compact structure for the *C. albicans* biofilm and single loosely located budding cells. Other pathological forms of yeasts are invisible. There was an apparent change in the *C. albicans* morphotype in the *S. mutans* common culture and visible pleomorphic forms were true hyphae, blastoconidia, which in the mixed culture also produce mycelial forms, whose role is related to damage to immune cells (macrophages) leading to microorganism invasion. There was abundant extracellular matrix between cells and covering bacterial and yeast cells. Bacterial cells were visible in chains adhering to yeast cells and wrapped around them. There was no clear, compact *S. mutans*/*C. albicans* biofilm structure or single loosely located budding cells. Other morphological forms of *C. albicans* were invisible. There was no visible extracellular matrix (**D**,**F**), which is formed by *S. mutans* alone and *S. mutans* with *C. albicans* treated with the *Lactobacillus salivarius* (**C**,**E**). Original magnification: 400×, 4000× and 6000×.

The inhibitory effect of *Lactobacillus salivarius* (HM6 Paradens) on biofilm generation by *S. mutans*, yeasts, and the co-culture is shown in Figure 3 and Figure 4, and the scanning electron microscope images (Figure 5B,D,F, Figure 6, Figure 7 and Figure 8B,D,F) after 24 h biofilm formation. The photographs show morphologically different fungal colonies and bacterial cells in single and co-cultures, before and after the administration of *Lactobacillus salivarius*.

The quantities of microorganisms’ (*S. mutans*, *C. albicans*, and the co-culture) logCFU/mL forming biofilms at 18, 20, 22, and 24 h were statistically lower after the administration of a probiotic in all analyzed groups (Figure 3, Figure 5 and Figure 6). The *p* value was statistically significant for 18–24 h points.

The median (range) bacterial count log(CFU/mL) value for single-species *S. mutans* biofilm after 24 h was 7.643 (7.568–7.756) and was significantly higher than the bacterial count log(CFU/mL) after probiotic administration: 7.505 (7.415–7.623) (Kruskal–Wallis test for dependent (repeated) measurements; *p* < 0.05) (Figure 3 and Figure 6).

The median (range) bacterial count log(CFU/mL) value for single-species *C. albicans* biofilm after 24 h was 6.279 (6.114–6.462) and was significantly higher than the bacterial count log(CFU/mL) after probiotic administration: 5.900 (5.833–5.991) (Kruskal–Wallis test for dependent (repeated) measurements; *p* < 0.05) (Figure 3 and Figure 6).

The median (range) bacteria/fungi count log(CFU/mL) value for double-species *S. mutans*/*C. albicans* biofilm after 24 h was 9.267 (9.041–9.477) and was significantly higher than the log(CFU/mL) after probiotic administration: 8.816 (8.633–8.940) (Kruskal–Wallis test for dependent (repeated) measurements; *p* < 0.05) (Figure 3 and Figure 6).

### 3.4. Analysis of Formed Biofilm Mass before and after Incubation with L. salivarius Probiotic

The total biomass (OD) of microorganisms (*S. mutans*, *C. albicans*, and the co-culture) producing a biofilm at 18, 20, 22, and 24 h were statistically lower after the administration of the probiotic in all the analyzed groups. The *p* value was statistically significant for all time points (18–24 h) (Table 3).

The inhibitory effect of *L. salivarius* (HM6 Paradens) on biofilm generation by *S. mutans*, yeasts, and the co-culture is shown in Table 3 and Figure 4 and Figure 7 after a 24 h biofilm formation. 

The mean biofilm mass (OD) value for single-species *S. mutans* biofilm after 24 h was 0.139 ± 0.007 and was significantly higher than the OD value after probiotic administration: 0.131 ± 0.004 (paired Wilcoxon’s test; *p* < 0.001) (Figure 4 and Figure 7).

The mean biofilm mass (OD) value for single-species *C. albicans* biofilm after 24 h was 0.100 ± 0.013 and was significantly higher than the OD value after probiotic administration: 0.084 ± 0.006 (paired Wilcoxon’s test; *p* < 0.001) (Figure 4 and Figure 7). 

The mean biofilm mass OD value for double-species *S. mutans*/*C. albicans* biofilm after 24 h were 0.176 ± 0.024 and was significantly higher than the OD value after probiotic administration: 0.127 ± 0.005 (paired Wilcoxon’s test; *p* < 0.001) (Figure 4 and Figure 7).

### 3.5. Relationship between the Colony Forming Unit Log(CFU/mL) and the Biofilm Mass (OD) before and after Lactobacillus salivarius Administration

**Figure 9:**
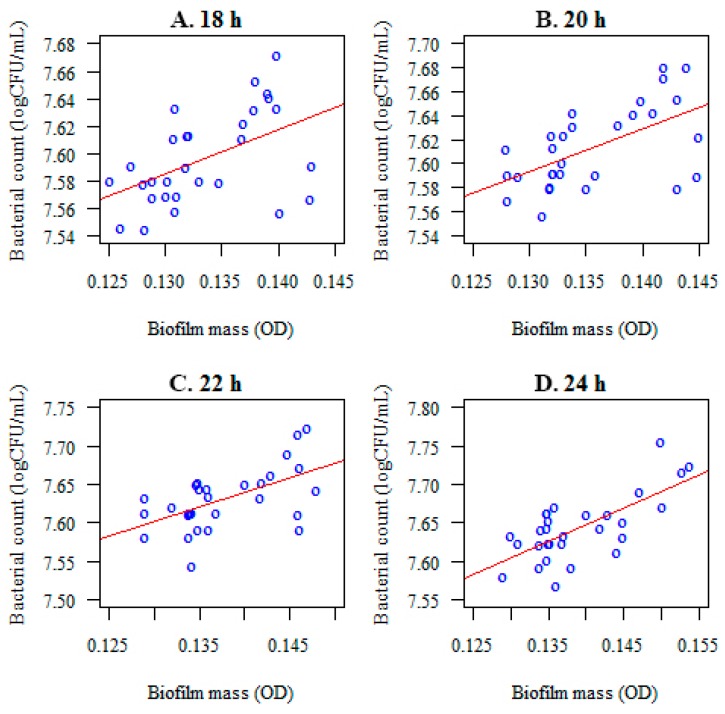
Correlations between the number of microorganisms forming a biofilm (*S. mutans*) (log(CFU/mL) and the optical density (biofilm mass) after 18 (**A**), 20 (**B**), 22 (**C**), and 24 (**D**) h of incubation, before *Lactobacillus salivarius* probiotic administration.

**Figure 10:**
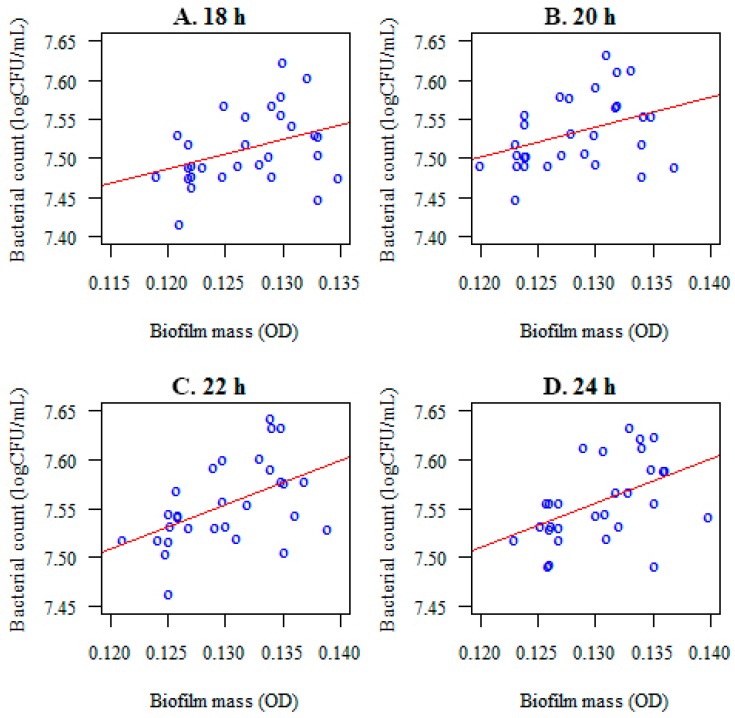
Correlations between the number of microorganisms forming biofilm (*S. mutans*) colony forming units and the optical density (the biofilm mass) at different time points ((**A**)–18 h; (**B**)–20 h; (**C**)–22 h; (**D**)–24 h) after *Lactobacillus salivarius* (HM6 Paradens) administration.

A correlation was noted between the microorganisms forming a biofilm (log(CFU/mL) and their mass (optical density) at the considered time points (Figure 9). After the administration of *Lactobacillus salivarius* (HM6 Paradens), the correlation between colony forming units and the *S. mutans*, the biofilm mass (OD) was lower or not present at all (Figure 10 and Table 4). For the *C. albicans* and *S. mutans*/*C. albicans* biofilms, no correlation between log(CFU/mL) and biofilm biomass (OD) was observed (Table 4).

The observed correlation was statistically significant throughout the biofilm generation (*p* < 0.05). The following connection was positive: the higher the number of colony forming units of tested microorganisms the higher optical density (biofilm mass). The strongest associations were reported after 24 h of biofilm generation (Table 4).

## 4. Discussion

The formation of biofilm on the surface of the teeth is a major factor in the development of early childhood caries (ECC) [1]. Studies have shown that bacteria and fungi present in biofilms may communicate with each other by sending extracellular signaling molecules or physical intercellular interactions to support the formation and development of cariogenic biofilms that contribute to the progression and recurrence of ECC [42].

The multifactorial etiology of the disease makes it necessary to perform a complex evaluation. The main causes include mutual host-microbial interactions, changes in mixed biofilms (reflecting both the richness of the microorganisms of the oral cavity and its conditions), and external factors initiating continuous pH changes in dental plaque. Stress factors associated with a reduction in the pH of the oral environment are responsible for the decreasing diversity of bacterial species that form oral biofilms. This results in minimization of metabolic activity (particularly, pyruvate kinase as the key enzyme for the entire process) and structural damage to the cell membrane, proteins, and DNA [43,44]. Hence, there are few species of microorganisms able to have an active metabolism under the above-mentioned conditions [45]. Dental plaque is associated with oral conditions associated with a drop in pH, resulting in the dominance of acid-producing strains connected to the development of caries. In physiological conditions, saprophytic/probiotic bacteria maintain the microbial balance and do not allow pathogenic flora to overgrow. The present paper describes the effect of a probiotic containing *L. salivarius* (HM6 Paradens) on a double-species biofilm of *S. mutans*/*C. albicans* as a new approach to maintaining the interspecies balance in the oral cavity and an additional method for supporting existing caries prevention methods.

A number of clinical studies have indicated that daily intake of *Lactobacillus*-derived dairy products can reduce caries, improve overall health, and reduce the need for antibiotic use in preschool children. However, not all children eat dairy products equally rich in probiotic substances. In addition, the impact of probiotics on oral health and caries inhibition in children is not entirely clear; further, their effects appear to be short-lived and are often directed exclusively at the reduction of cariogenic *S. mutans*, without affecting other microbial species colonizing the oral cavity that have been confirmed to contribute to caries development. 

As a result of advanced technology, the number of documented microorganism species involved in the development of caries is increasing. There are literature data indicating that, in addition to *S. mutans* and *C. albicans*, fungi are involved in the carious process and are detectable in large amounts in the plaque and saliva of children with ECC [1,26,45,46,47].

Bacterial–fungal infections are common in humans, as part of the natural physiological flora. Under favorable conditions, microorganisms that have been thus far regarded as saprophytes incapable of inducing human diseases and have become an etiological factor in a number of diseases [3,48]. *C. albicans*, as a main representative of a fungal microbiome, is present in the mouth, in the mucous membranes, dentures, and orthodontic devices [49,50]. However, a number of observations have indicated that interactions between yeasts and oral streptococci can also occur on cleansed surfaces of enamel, dentine, or on the surface of dental plaque [51,52], particularly in the presence of nutrients, such as sucrose [53,54].

We demonstrated that in the presence of a probiotic, containing inactivated *L. salivarius* (HM6 Paradens), biofilm formation was different from that in a double-species model, without the use of the probiotic. After a suitable culture time, there was no observable mixed *S. mutans* and *C. albicans* biofilm formation. Single aggregates of yeasts and streptococci did not produce a common structure, as in the double-species model. This suggests that *L. salivarius* perhaps competes with *S. mutans* for nutrient substrates and does not allow these bacteria to consume them, along with inhibiting the aggregation of oral streptococci and yeasts, thus resulting in the lack of a double-species biofilm structure. It is necessary to carry out studies that would either provide or deprive biofilms of certain nutrients, in order to confirm the hypothesis of a possible mechanism.

In this study, CFU/mL results showed a higher number of *C. albicans* in mixed *C. albicans/S. mutans* biofilms, compared to single-species *C. albicans* biofilms, indicating that *S. mutans* stimulate *C. albicans* growth. These results coincide with those of Júnia Oliveira Barbos et al. and Tomé et al. [55,56]. Although the molecular mechanism for these behaviors is undefined, He et al. [57] confirmed that the interaction between *S. mutans* and *C. albicans* is related to the upregulation of most carbohydrate-transport-linked genes and metabolic processes. The presence of *C. albicans* enhances the expression of 393 genes in *S. mutans* in the double-species biofilm, as compared to the single *S. mutans* biofilm. Molecular studies have shown that the coexistence of *S. mutans* with *C. albicans* affects the use of carbohydrates by *S. mutans*. Furthermore, a co-culture with *C. albicans* changes the transcription of *S. mutans* signal transduction genes (comC and ciaRH) associated with its condition and virulence [3]. Occurrence of a biofilm at this stage may depend on the *C. albicans* morphotypes showing a twofold nature: buds and *C. albicans* hyphae may colonize mucous membranes and constitute physiological microflora (commensal) or may lead to infection under favorable conditions (opportunistic pathogens). These data provide extensive evidence for bacterial–fungal interactions that may progress to dental caries or recurrence of the disease in the future. 

During initial adhesion of pioneer colonizers, such as *S. mutans*, to an enamel surface, there is increased activation of glucosyltransferases (Gtfs), particularly GtfB. These, in turn, become the binding sites of secondary colonizers, such as *C. albicans* and *S. mutans* [58]. Fungi, occurring in a hyphal form [59], exhibit better adhesion to the surface than blastospores. However, these effects are not as strong as when *C. albicans* is present in association with *S. mutans* [24], as stated in the literature. This is probably related to increased levels of proteolytic enzymes, i.e., aspartyl proteinases (Saps) of *C. albicans*, which increase mutual interspecies interactions. In particular, Sap1–5 dominate in dental biofilms and can be a crucial factor in ECC development [60,61]. These, and a number of other, yet unknown, unique effects enhance the adhesion properties of both microorganisms for teeth colonization.

In terms of the double-species *S. mutans/C. albicans* biofilm, one can note that fungi occurring in the form of hyphae and blastoconidia are much better at biofilm-producing than in the absence of these forms, under the action of the *L. salivarius* probiotic (HM6 Paradens), as seen in scanning electron microscope images (Figure 4C,D). This formulation can act as a proteolytic enzyme inhibitor, i.e., aspartyl proteases (Saps) of *C. albicans*, which are considered to be the main contributor to interspecies interactions. The above observation is quite innovative, since research on *S. mutans* and *C. albicans* biofilms has confirmed that *S. mutans* stimulates *C. albicans* to grow as biofilms in vitro. However, it has been thought so far that the bacteria inhibit the formation of hypha by yeast [51]. In our studies on clinical strains from children with childhood caries, the opposite effect can be observed. In SEM samples, double-species biofilms were plentiful in hyphae formed by fungi, which was not noticeable after a probiotic application. When evaluating the fungal morphology, we noticed that *L. salivarius* inhibited the formation of germ tubes and pseudomycelium when added to the *S. mutans* and *C. albicans* common culture. These observations indicate that this probiotic may produce signaling molecules or indirectly inhibit the generation of double-species biofilm by inhibiting *S. mutans*. Without the use of a probiotic, *S. mutans* supported the cariogenicity of the biofilm. In the presence of HM6, *C. albicans* mycelium was not formed in a mixed biofilm. The present model seems interesting, due to the fact that, thus far, it has been presumed that it is *S. mutans* in the *C. albicans* mixed biofilm that sends signaling molecules, such as mutanobactin A, that naturally inhibit *C. albicans hyphae*, thus preventing the development of cariogenic biofilms.

This results in increased production of the EPS biofilm matrix and a specific hyperadditive effect that enhances the biofilm virulence. *C. albicans* dominates in the competition for unique eco-niches, such as teeth gaps and fractures [62], and, using its natural ability for thigmotropism, penetrates deep into the open dental canals. Owing to increased penetration of hard-to-reach places, such as tooth roots, *C. albicans* leads to aggressive caries and contributes to rapid progression of the disease [63,64]. It is necessary to further study the mechanisms of multispecies interactions of yeasts and oral streptococci in the biofilms during which EPS growth and the activation of metabolic pathways has a significant impact on the aggressiveness of the formed structure and the rapid progression of the disease. The results of this study explain why current caries prophylaxis, based only on *S. mutans* identification, is not as effective as screening methods for diagnosing children at risk for early caries. Research should be conducted on the mutual relationships between potentially interacting microorganisms (pathogenic and non-pathogenic). An approach, based on the current state of knowledge on caries, increases the chances of understanding the pathogenesis of this disease and may, therefore, lead to new ways of prevention. Then, it will be possible to design potential modulators for the development and progression of diseases, such as caries.

The studies on the *S. mutans/C. albicans* interactions cited earlier in this work concerned biofilm models using in vitro reference strains. The presented model uses clinical strains and shows the variability that clinical strains may exhibit, even within the same species. The same strain does not necessarily induce a hostile response to disease development. Calculation of the pathogenic effect for microorganisms appears to be estimative and despite an inoculum-dependent effect, there are still other limitations, such as the occurrence of variable conditions of infection [65]. Comparisons of virulence among microorganisms provide evidence that any action aimed at eliminating its factors should be undertaken with caution, given that any change in the host–microbial relationship can alter the pathogenic potential of microorganisms.

Although in vivo studies seem to be much more reflective of the real situation, they are becoming increasingly limited due to ethical reasons. 

Application of an in vivo model, using potentially cariogenic clinical strains, and evaluation of the effect of *Lactobacillus salivarius* (HM6 Paradens) probiotic on *C. albicans*/*S. mutans* co-culture biofilm were the objectives of this study.

This observation demonstrates that a probiotic containing thermally inactivated *L. salivarius* strains inhibited the ability of the strains to form a common structure between oral streptococci and yeasts. The examined strains were derived from the dental biofilm of children with early childhood caries. The evaluation of the generated biofilm under the influence of the test compound showed a decrease in both the grown colonies and the biofilm biomass, along with reduced cross-linking of the biofilm structure. These results coincide with those of Wu et al. [66], Ahmed et al. [67], and Nishihara et al. [15].

The obtained results are of interest because a number of studies using probiotics appear to be controversial, particularly as probiotics that inhibit the growth of *S. mutans* monocultures do not necessarily reduce the cariogenicity of multi-species biofilms [68], where *S. mutans* is only one component among a number of other plaque-forming species [69]. Therefore, it seems more appropriate to study the effect of probiotics on the relationships between the different biofilm-forming species and the reduction of cariogenic *S. mutans*. In addition, the above data on the inhibition of biofilm formation, not only of *S. mutans* but also by the *S. mutans*/*C. albicans* co-culture, indicate that the action of the *Lactobacillus salivarius* (HM6 Paradens) is very promising. It meets the modern definition of caries as a multifactorial disease [70], which is dependent on a number of species of microorganisms forming biofilms and not just on selected single species of bacteria [71]. Furthermore, the findings support the assumption of Koo and Bowen [72], who proposed the possibility of including anti-fungal (anti-*Candida*) therapy for ECC.

Although there are many published studies showing the mutual interactions of fungi with bacteria, including *S. mutans*, most of the mechanisms responsible for these processes remain unclear. Recently, several key findings have emerged that characterize the molecules involved in the *S. mutans/C. albicans* interactions. However, these models do not take into account the factors connected to the host and pathogenicity of the strains depending on the host environment. It is known that under certain conditions, bacteria or fungi acquire features that condition their pathogenicity [65]. Our model has the advantage of taking into account clinical strains from the source of infection (dental plaque from children with caries), which have developed their virulence in response to the environment where they exist. This is confirmed by the results on interactions between *S. mutans* and *C. albicans* clinical strains, which produce pathogenic factors such as mycelia and germ tubes in the co-culture. This is constrast to the results obtained by other researchers (using standard strains), where such factors were not present. International research uses advanced technology, but such technology is not always or everywhere accessible and applicable. Thus, the observations obtained during the presented studies are unique because they show changes in the level of fungal morphogenesis associated with the production of mycelium. In clinical practice, this is a manifestation of *C. albicans* pathogenesis, associated with an active infection, which is severe caries. Continuing the observed relationships, in the future we would like to evaluate hyphal growth factor HWP1, or ALS1 and ALS3, which, as seen in studies by Ellepola et al. [42], may be a potential mechanism of mycelial growth in *S. mutans/C. albicans* co-cultures. We plan to verify how available nutrients, except sucrose, modify the above-mentioned feature. This new discovery may explain the mutual roles of *S. mutans* and *C. albicans* in cariogenic biofilm formation and provide a point of action for the creation or utilization of molecules with antineoplastic potential. The results of the study on the inhibition of cariogenic *S. mutans/C. albicans* biofilm are also a prerequisite for further research on this probiotic in in vivo studies. In addition, the multifunctional nature of biofilm development and resistance to conventional treatment encourage the use of probiotics, which are intended to protect native microflora by interfering with microorganisms accumulated in the biofilm structure.

Probiotic bacteria, such as *L. salivarius*, colonize the oral cavity of naturally born children [73]. Their protective role may be based on the stimulation of the host’s immune system to produce antibodies and immunoglobulins [74]. The results of the potential use of probiotics in caries are very encouraging. Our study, although a small section of probiotic research, encourages us to take further steps to establish a consensus on the use of probiotics in the prevention of oral diseases, such as dental caries. The research is important, as the available databases have only a few clinical studies with strong scientific evidence proving the effectiveness of probiotics in ECC counteraction [64].

## 5. Conclusions

Research on the use of probiotics in the prevention of oral diseases, such as dental caries, has opened up new opportunities for establishing a balance between diet and oral health. Studies show that probiotic bacteria given in any form are safe for humans and can provide a good complement to daily oral hygiene. Administering probiotics based on the occurrence of a natural comorbid flora in our organism is the future of biotherapeutic research, as these bacteria are ideally adapted to the human microbiome and occupy the supreme position while displacing potentially pathogenic species. Probiotic supplementation to a daily balanced diet may be a strategy for preventing caries or other oral infectious diseases in children.

## Figures and Tables

**Table 1:** Division of study groups according to patient age.

Examined Groups	Age: 3 Years (*n* = 6)	Age: 4 Years (*n* = 20)	Age: 5 Years (*n* = 28)	Age: 6 Years (*n* = 5)	*p* *
	*n*	*n*	*n*	*n*	
Non-cavitated	2	11	13	4	0.489
Cavitated	4	9	15	1	

* Fisher’s exact test (because of the low numbers in the table).

**Table 2:** Frequency of isolation of *S. mutans* and yeasts in the studied groups of patients.

Microorganisms	Girls (*n* = 25)	Boys (*n* = 34)	Total (*n* = 59)
	*n*	*n*	*n*
*C. albicans*	12	18	30
*S. mutans*	13	16	29

**Table 3:** *S. mutans*, *C. albicans*, and oral streptococci/yeast biofilm formation: total biomass (OD) evaluation before and after administration of *Lactobacillus salivarius* (HM6 Paradens, LS) at individual time points.

Species	Time	Test	*n*	Mean	SD	Median	Min	Max	Q1	Q3	*p* ***
***S. mutans***	18 h	Before LS	29	0.134	0.005	0.132	0.125	0.143	0.130	0.138	<0.001
		After LS	29	0.127	0.005	0.127	0.119	0.135	0.122	0.130	
	20 h	Before LS	29	0.136	0.005	0.134	0.128	0.145	0.132	0.141	<0.001
		After LS	29	0.128	0.005	0.128	0.120	0.137	0.124	0.132	
	22 h	Before LS	29	0.138	0.006	0.136	0.129	0.148	0.134	0.143	<0.001
		After LS	29	0.130	0.005	0.130	0.121	0.139	0.126	0.134	
	24 h	Before LS	29	0.139	0.007	0.136	0.129	0.154	0.135	0.144	<0.001
		After LS	29	0.131	0.004	0.131	0.123	0.140	0.126	0.134	
***C. albicans***	18 h	Before LS	30	0.095	0.013	0.094	0.070	0.120	0.084	0.106	<0.001
		After LS	30	0.078	0.006	0.080	0.070	0.092	0.074	0.082	
	20 h	Before LS	30	0.098	0.013	0.097	0.072	0.123	0.086	0.108	<0.001
		After LS	30	0.081	0.006	0.082	0.072	0.094	0.076	0.084	
	22 h	Before LS	30	0.100	0.013	0.100	0.074	0.125	0.088	0.110	<0.001
		After LS	30	0.083	0.006	0.084	0.074	0.096	0.078	0.086	
	24 h	Before LS	30	0.100	0.013	0.101	0.075	0.126	0.089	0.111	<0.001
		After LS	30	0.084	0.006	0.085	0.074	0.097	0.080	0.086	
***S. mutans/C. albicans***	18 h	Before LS	29	0.171	0.024	0.179	0.125	0.221	0.151	0.191	<0.001
		After LS	29	0.123	0.006	0.122	0.112	0.131	0.118	0.129	
	20 h	Before LS	29	0.173	0.024	0.181	0.127	0.224	0.154	0.193	<0.001
		After LS	29	0.125	0.006	0.124	0.114	0.134	0.120	0.130	
	22 h	Before LS	29	0.175	0.024	0.183	0.129	0.226	0.156	0.195	<0.001
		After LS	29	0.127	0.005	0.126	0.115	0.134	0.123	0.132	
	24 h	Before LS	29	0.176	0.024	0.184	0.130	0.228	0.157	0.194	<0.001
		After LS	29	0.127	0.005	0.127	0.117	0.134	0.123	0.132	

Wilcoxon’s test was used for dependent (repeated) measurements (paired Wilcoxon’s test; * *p* < 0.05, *** *p* < 0.001). SD, standard deviation.

**Table 4:** Correlations between the number of microorganisms forming a biofilm (*S. mutans*, *C. albicans*, and *S. mutans/C. albicans*) and the optical density (biofilm mass, OD) at different time points, before and after *Lactobacillus salivarius* (HM6 Paradens, LS) administration.

Species	Incubation Time	Before LS		After LS	
		*r*	*p*	*r*	*p*
***S. mutans***	18 h	0.495	0.006	0.371	0.048
	20 h	0.534	0.003	0.379	0.043
	22 h	0.500	0.006	0.502	0.005
	24 h	0.553	0.002	0.473	0.009
***C. albicans***	18 h	0.920	<0.001	0.136	0.473
	20 h	0.918	<0.001	0.128	0.927
	22 h	0.931	<0.001	0.127	0.888
	24 h	0.935	<0.001	0.135	0.853
***S. mutans/C. albicans***	18 h	0.764	<0.001	0.181	0.337
	20 h	0.769	<0.001	0.191	0.393
	22 h	0.766	<0.001	0.200	0.459
	24 h	0.842	<0.001	0.192	0.628
